# Zebrafish Seizure Model Identifies *p*,*p′*-DDE as the Dominant Contaminant of Fetal California Sea Lions That Accounts for Synergistic Activity with Domoic Acid

**DOI:** 10.1289/ehp.0901301

**Published:** 2009-12-04

**Authors:** Jessica A. Tiedeken, John S. Ramsdell

**Affiliations:** Marine Biotoxins Program, Center for Coastal Environmental Health and Biomolecular Research, National Oceanic and Atmospheric Administration, National Ocean Service, Charleston, South Carolina, USA

**Keywords:** Danio rerio, DDT, domoic acid, epilepsy, PBDE, PCB, seizures, zebrafish

## Abstract

**Background:**

Fetal poisoning of California sea lions (CSLs; *Zalophus californianus*) has been associated with exposure to the algal toxin domoic acid. These same sea lions accumulate a mixture of persistent environmental contaminants including pesticides and industrial products such as polychlorinated biphenyls (PCBs) and polybrominated diphenyl ethers (PBDEs). Developmental exposure to the pesticide dichlorodiphenyltrichloroethane (DDT) and its stable metabolite 1,1-bis-(4-chlorophenyl)-2,2-dichloroethene (*p,p′-*DDE) has been shown to enhance domoic acid–induced seizures in zebrafish; however, the contribution of other co-occurring contaminants is unknown.

**Objective:**

We formulated a mixture of contaminants to include PCBs, PBDEs, hexachlorocyclohexane (HCH), and chlordane at levels matching those reported for fetal CSL blubber to determine the impact of co-occurring persistent contaminants with *p,p′*-DDE on chemically induced seizures in zebrafish as a model for the CSLs.

**Methods:**

Embryos were exposed (6–30 hr postfertilization) to *p,p′*-DDE in the presence or absence of a defined contaminant mixture prior to neurodevelopment via either bath exposure or embryo yolk sac microinjection. After brain maturation (7 days postfertilization), fish were exposed to a chemical convulsant, either pentylenetetrazole or domoic acid; resulting seizure behavior was then monitored and analyzed for changes, using cameras and behavioral tracking software.

**Results:**

Induced seizure behavior did not differ significantly between subjects with embryonic exposure to a contaminant mixture and those exposed to *p,p′*-DDE only.

**Conclusion:**

These studies demonstrate that *p,p′*-DDE—in the absence of PCBs, HCH, chlordane, and PBDEs that co-occur in fetal sea lions—accounts for the synergistic activity that leads to greater sensitivity to domoic acid seizures.

California sea lions (CSLs; *Zalophus californianus*) are subject to multiple classes of environmental stressors, including exposure to persistent environmental contaminant burdens, infection by several pathogens, and episodic poisoning by toxins from harmful algal blooms (DeLong et al. 1973; Gerber et al. 1993; Scholin et al. 2000). It has been proposed that exposure to these different classes of stressors contributes to reproductive failure events in this species (Brodie et al. 2006; Gilmartin et al. 1976). This and similar unusual mortality/morbidity trends or events provide leads toward identifying environmentally relevant stressor interactions in natural populations (Greig et al. 2005). The last four decades of investigations into reproductive failure of the sea lion population in the California Channel Islands National Marine Sanctuary (CINMS) has revealed exposure to a mixture of stressors, including environmental contaminants [dichlorodiphenyltrichloroethanes (DDTs) and polychlorinated biphenyls (PCBs)] and disease (leptospirosis and San Miguel sea lion virus), along with the rising presence of the algal toxin domoic acid (Greig et al. 2005). Research encompassing the complexity of potential stressors that converge by happenstance at a time or place of interest is referred to as “coincidental mixtures” and is considered among the most difficult areas for environmental health research (Sexton and Hattis 2007).

One component of these stressors, environmental chemical contaminants, has been documented in CINMS sea lion cows and found to be transferred to their fetuses through the placenta (Greig et al. 2007). The most abundant of these compounds, 1,1-bis-(4-chlorophenyl)-2,2-dichloroethene (*p,p′*-DDE), has been described by food web bioaccumulation and physiologically based pharmacokinetic models to increase in the fetus during the course of development (Connolly and Glaser 2002; You et al. 1999). Another environmental stressor, the algal toxin domoic acid, causes abortion, premature parturition, or death of pregnant female sea lions and readily permeates the placenta to accumulate in amniotic fluid and poison the fetus (Brodie et al. 2006; Maucher and Ramsdell 2007). The seasonality of massive algal blooms in the vicinity of the CINMS during the late spring upwelling periods places domoic acid exposure at the end of gestation for the CSL and at completion of neurodevelopment of the fetus (Ramsdell and Zabka 2008). This coincidence of two stressors, *p,p′*-DDE and domoic acid, in fetal sea lions—each known to promote neurodevelopmental toxicity—led to a testable exposure scenario for coincidental mixtures.

We previously reported that *p,p′-*DDE exposure of zebrafish embryos increases the sensitivity and the manifestation of seizure behavior induced by domoic acid after brain maturation (Tiedeken and Ramsdell 2009). The body concentration of *p,p′-*DDE in zebrafish at the time of domoic acid exposure corresponded to the upper range found in fetal sea lions near term (Tiedeken and Ramsdell 2009). Given the complex body burden of persistent contaminants such as PCBs, polybrominated diphenyl ether (PBDEs), and persistent pesticides in addition to DDTs in fetal sea lions, a compelling question is whether other dominant contaminant components of these groups contribute to the *p,p′*-DDE effect to enhance domoic acid–induced toxicity. To test this question, we exposed zebrafish embryos to *p,p′*-DDE in the absence and presence of mixtures formulated to match contaminant composition in fetal sea lion blubber, as recently determined by Goldstein et al. (2009). We then analyzed induced seizure behavior in the zebrafish after brain maturation.

## Materials and Methods

### Zebrafish

Fifteen breeding pairs of AB wild-type strain zebrafish (*Danio rerio*) obtained from Zebrafish International Resource Center (Eugene, OR) were bred at random to obtain embryos. Fish were kept in a recirculating aquarium rack system (Aquatic Habitats, Apopka, FL) with environment maintained according to the manufacturer’s protocol and *The Zebrafish Book* (Westerfield 2000). Embryos that fell through the breeding insert were collected within an hour after lights came on and were washed with sterile tank water. The zebrafish and offspring were handled humanely and euthanized when we were unable to otherwise ameliorate distress.

### Contaminant mixture formulations

The environmental contaminants β-hexachlorocyclohexane (β-HCH), all PCBs, PBDE-47, and *trans*-nonachlor were obtained from ChemService Inc. (West Chester, PA) in dried standard neat form. The additional PBDEs 99, 100, and 28, which were obtained from AccuStandard (New Haven, CT), arrived as standards in isooctane and were dried under nitrogen. All other chemicals were purchased from Sigma Chemical Company (St. Louis, MO). *p,p′*-DDE and the other contaminants were resuspended and diluted in dimethyl sulfoxide (DMSO).

We prepared contaminant formulations to desired concentrations so that the contaminants were at ratios corresponding to average levels reported in blubber of 14 premature sea lion pups described by Goldstein et al. (2009). All contaminant formulations were based relative to a standard dose of *p,p′*-DDE (1 μM), which was previously determined to enhance sensitivity to chemical convulsants and also to accumulate to levels in zebrafish comparable with those in fetal sea lions (Tiedeken and Ramsdell 2009).

### Contaminant screening using a bath exposure protocol

To test potential synergy with chemical convulsants, we administered a formulation of primary components, which included the most prevalent compounds across groups of contaminants found in fetal sea lions (PCB-138, PCB-153, β-HCH, PBDE-47, and *trans*-nonachlor) via bath exposure (Table 1). The contaminant composition was adjusted proportionately and added to a 1-mM *p,p′*-DDE standard or 2-mM *p,p′*-DDE concentration to verify effect with a dose response (Table 1). We used a DMSO working stock of 1,000× desired test concentrations (Table 2), resulting in 0.1% DMSO (vehicle) and micromolar contaminant levels in zebrafish water exposure. Embryos, in “shield” stage [6 hr postfertilization (hpf)], were treated in a 6-well plate with 1 μL of the contaminant formulation stock per milliliter of zebrafish water, at a density of 10 embryos/mL. After 24 hr exposure, the contaminant water was replaced with clean media and embryos were nurtured until the chemical convulsant trial, as described previously (Tiedeken and Ramsdell 2009).

### Complex contaminant administration via microinjection

We used microinjection directly into the yolk sac to treat zebrafish embryos *in ovo* with a formulation containing more complete components of persistent contaminant mixtures (Table 1). We chose microinjection over bath application to minimize any differences in uptake of individual contaminants by the embryo and to ensure that the less-dominant components would also reach the yolk. We used a 1-nL injection of DMSO as the microinjection vehicle because this was the largest volume that had no interfering effects on embryos. The small volume was formulated based on the amount (7.499 ng) of *p,p′*-DDE detected per larvae after a 1-μM bath exposure (Tiedeken and Ramsdell 2009); concentrations are listed in Table 3. This complex formulation was once again doubled in concentration, not volume, to verify effect with a dose response. The high starting concentration of *p,p′*-DDE impeded flow through the microinjection needles as it passed into the embryo and was subsequently excluded from the injection formulation and administered in the aqueous bath (Table 1).

Embryos, < 5 hpf, were stabilized in troughs on agarose plates and covered with 12.5% Hank’s Solution (Westerfield 2000). Needles were handcrafted and beveled using Sutter pipette puller (P-87) and beveller (BV-10) (Sutter Instrument Co., Navato, CA). We adjusted pressure on a nitrogen gas pico injector (PLI-100; Harvard Apparatus, Holliston, MA) to achieve a 1-nL drop, which was injected into the embryo yolk. After injection, embryos were allowed to recover in the plate before being transferred to *p,p′*-DDE bath treatments as described earlier at 6 hpf. Controls were injected with vehicle (DMSO) before exposure to 0.1% DMSO bath. Those exposed to *p,p′*-DDE only were microinjected with DMSO to negate any potential effects from the microinjection procedure. Embryos injected with the contaminant formulation were exposed only to the corresponding *p,p′*-DDE bath concentration (Table 1).

### Analysis of pentylenetetrazole (PTZ)- or domoic acid–induced seizure behavior

At 7 days postfertilization (dpf), after having undergone completion of brain formation at 5 dpf, zebrafish exhibit behavioral, electrographic, and molecular changes to chemical convulsants that would be expected from a rodent seizure model. Seizure behavior was induced with either 5 mM PTZ or 0.36 mM domoic acid in Ringers solution (Westerfield 2000) administered 7 dpf, as described previously (Tiedeken and Ramsdell 2009). We used PTZ, a competitive antagonist of the GABA-A (gamma-aminobutyric acid type A) receptor, as an initial convulsant test agent to conduct a high-throughput screening of seizure behaviors that has been developed as a seizure model (Baraban 2006). An effective 5-mM concentration was administered to 30 embryos per treatment group in a 96-well plate setup, and behaviors were recorded for at least 20 min in accordance with methods described previously (Baraban et al. 2005; Tiedeken and Ramsdell 2007, 2009). Domoic acid, an agonist of AMPA (α-amino-3-hydroxyl-5-methyl-4-isoxazole-propionate) and kainate subtypes of glutamate receptors, was used to create a seizure challenge relevant to the CSL at a concentration of 0.36 mM. Ten larvae per treatment group were placed individually into 96-well plates for the challenges, and behavior was recorded for at least 20 min. All larvae were euthanized after the observation period with a lethal dose of MS-222 (tricaine methanesulfonate).

We anlyzed PTZ-induced seizure behaviors using EthoVision (Noldus Information Technology, Leesburg, VA) tracking software; larvae were monitored for increased movement (stage I), circular swimming (stage II), and convulsive behaviors (stage III). Domoic acid–induced seizure behavior was scored manually, with convulsions, twitches, and other movements noted and classed according to severity. Erratic swimming and twitching behaviors were grouped as class 1, convulsions and associated behaviors defined as class 2, and loss of posture and paralysis were the major components of class 3 behaviors. Both types of seizure behavior were measured and analyzed using scoring characteristics and seizure classifications described by Baraban et al. (2005) and Tiedeken and Ramsdell (2009). We used one-way analysis of variance followed by Dunnett’s means comparison test to analyze EthoVision-measured parameters that were normalized against a baseline.

## Results

### Effect of primary contaminant formulation on induced seizure behavior

**Figure 1:**
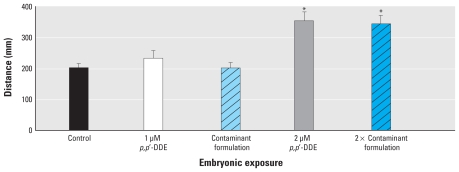
Distance traveled (mean ± SE) after exposure to 5 mM PTZ grouped by embryonic bath pretreatments to *p,p′-*DDE alone and in a contaminant formulation. *p < 0.01 compared with control.

Zebrafish embryos bath-exposed to a primary contaminant formulation in aqueous media (0.1% DMSO) during primary neurodevelopmental stages (6–30 hpf) showed no difference in effects compared with embryos exposed to *p,p′*-DDE alone. Morphologic development and viability in treated embryos were comparable with nontreated embryos, showing the contaminants had no detrimental effects on embryo survivability. When these exposed embryos grew into neurologically developed larvae (7 dpf), they were challenged with the seizure-inducing agent PTZ, and seizure behavior differences emerged. A PTZ-induced seizure is characterized in three stages: stage I, erratic movement; stage II, rapid circular swimming; stage III, loss of posture and body convulsions (Baraban et al. 2005). We noted significant changes in distance, demonstrating increased stage I and stage II seizure behaviors, between embryonic exposure to 1 μM *p,p′*-DDE and 2 μM *p,p′*-DDE (*p* < 0.01). However, the presence of additional contaminants did not significantly (*p* > 0.05) enhance distance traveled more than the *p,p′*-DDE alone (Figure 1).

**Figure 2:**
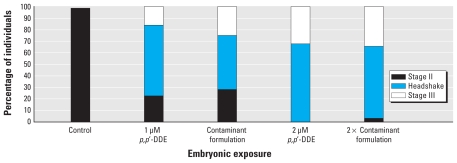
Percentage of individuals with the highest level of expressed seizure behaviors after exposure to 5 mM PTZ at 7 dpf following embryonic bath pretreatments to *p*,*p′-*DDE alone and in a contaminant formulation.

In a previous study (Tiedeken and Ramsdell 2009), embryonic exposure to *p,p′*-DDE resulted in a unique head-shake behavior when challenged with the seizure-inducing agents PTZ and domoic acid. In the present study we observed this characteristic head-shake behavior across all groups with embryonic *p,p′*-DDE pretreatment in the presence of PTZ. The increased concentration of *p,p′*-DDE pretreatment showed a 20% increase in the number of individuals with head-shake behavior and expression of more severe convulsive behaviors (Figure 2). The presence of the other primary contaminants alongside the *p,p′*-DDE pretreatment did not increase the severity of the seizure behaviors beyond the levels of *p,p′*-DDE alone.

Larval challenges to the marine toxin domoic acid generate a different pattern of seizure behaviors than observed with PTZ (Tiedeken and Ramsdell 2009). These behaviors were grouped in classes of perceived severity, with class 1 including erratic swimming and jaw movements, class 2 including convulsive behaviors and resulting movements, and class 3 including loss of posture and paralysis. When contaminant-treated embryos were challenged with domoic acid in the larval stage, the characteristic *p,p′*-DDE–induced head-shake behavior still emerged and was classified with the class 2 behaviors. This allowed for *p,p′*-DDE–pretreated embryos to exhibit twice as many class 2 instances and have 20% more individuals responding compared with vehicle-pretreated embryos (Table 4). In the larvae receiving the higher concentration of *p,p′*-DDE (2 μM), 100% of larvae responded and experienced an increase in class 2 and class 3 instances. Although this noticeable increase in severity and number of seizures related to the *p,p′*-DDE concentration in the pretreatment, the presence of other prominent contaminants (PCBs, PBDE, etc.) did not change the amount or types of seizure behaviors observed.

### Effect of complex contaminant formulation on induced seizure behavior

**Figure 3:**
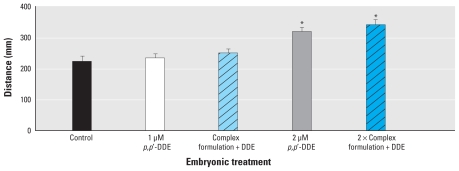
Distance traveled (mean ± SE) after exposure to 5 mM PTZ, grouped by embryonic pretreatments to microinjected complex contaminant formulation and *p,p′-*DDE. *p < 0.01 compared with control.

**Figure 4:**
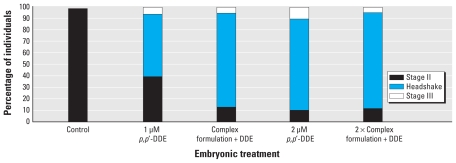
Percentage of individuals with the highest level of expressed seizure behaviors after exposure to 5 mM PTZ at 7 dpf following embryonic pretreatments to microinjected complex contaminant formulation and *p,p′-*DDE.

With the amount of contaminant uptake by embryos uncertain, especially because most of the contaminants were < 10% of total contaminant mass, we used microinjection to create consistency between uptake of individual contaminants. Microinjection also allowed us to use additional contaminants present at much lower levels. The complex contaminant formulation in 1-nL DMSO injected into the embryos caused no outward morphologic changes present in the 7 dpf larvae. When these larvae were challenged with 5 mM PTZ, the *in ovo* microinjection with DMSO had no effect on seizure parameters. Similar to previous PTZ seizure challenges, distance traveled in the well increased significantly (*p* < 0.01) with higher embryonic exposure to *p,p′*-DDE (2 μM) (Figure 3), but we found no significant difference (*p* > 0.05) between those injected with the complex contaminant formulation and those injected with vehicle alone. The mobility parameter also followed the same trend, with increases in response to the *p,p′*-DDE regardless of the presence of other environmental contaminants during development (data not shown). Head-shake behaviors were present in addition to or in place of a stage II seizure with preexposure to *p,p′*-DDE; however, this behavior was not modified with injection of the complex contaminant mixture (Figure 4). The percentage of animals exhibiting head-shake behavior did increase by 25% between the 1 μM *p,p′*-DDE alone and the complex contaminant formulation with 1 μM *p,p′*-DDE. This trend did not follow between exposures to 2 μM *p,p′*-DDE and the 2× complex contaminant formulation and was not reproduced in other measured parameters.

The exposure of contaminant-injected larvae to domoic acid correlates more closely with wildlife exposures, including both the embryonic presence of contaminants and a later exposure to domoic acid. Larval cohorts pretreated with *p,p′*-DDE experienced increased seizure behavior as noted by instances of class 2 and class 3 behaviors and the percentage of individuals responding, which continued to increase with higher *p,p′*-DDE concentrations (Table 5). This effect was attributed in part to the head-shake behavior in *p,p′*-DDE larvae, grouped as a class 2 severity seizure. Presence of the contaminants once again showed no noticeable increase of the domoic acid–induced seizure behavior, with similar quantity of responses and percentage of individuals responding between groups (Table 5).

## Discussion

### Formulated mixture for fetal sea lion contaminants

We previously reported that exposure to DDT or its metabolite DDE during zebrafish neurodevelopment enhances seizure behavior response to the algal toxin domoic acid after completion of brain maturation, a likely exposure scenario for the CSL population of the Channel Islands (Tiedeken and Ramsdell 2009). In the present study we have expanded upon this finding to evaluate the influence of *p,p′*-DDE in the presence of co-occurring persistent contaminants. We formulated a mixture of organochlorine pesticides, PCBs, and PBDEs in defined proportion to the single component (*p,p′*-DDE) that we previously determined to enhance chemical-induced seizures after completion of brain development. Investigating the interaction of PCBs with *p,p′*-DDE is especially relevant to the CINMS sea lions whose increased body burdens of DDTs were correlated with prenatal mortality events (DeLong et al. 1973), but the co-occurrence of elevated burdens of PCBs and other stressors precluded firm conclusion of the adverse health effect of any contaminant (Gilmartin et al. 1976). To investigate this potential interaction, we formulated the contaminant mixtures to match levels determined in 14 premature CSL pups sampled during a domoic acid–associated mortality event in 2005 on San Miguel Island (Goldstein et al. 2009).

The reconstituted mixture approach used here is based on the concentration in the fetal CSL that reflects transplacental transfer. Transplacental transfer of contaminants has been determined for both preterm and term CSLs and is best described by maternal load and fetal fat content (Greig et al. 2007). Accordingly, we used levels measured in preterm CSL fetuses, a life stage in which domoic acid poisoning commonly occurs. Our experimental approach was 2-fold. In the first test we used the most predominant contaminants—PCB-153 and PCB-138 (which represented up to 46% of total PCBs), β-HCH (100%), PBDE-47 (83%), and *trans*-nonachlor (80%)—formulated in proportion to levels reported for fetal CSL blubber. Finding no interactive effect with *p,p′*-DDE, we next increased the number of PCBs to a total of 20 congeners (to reach 95% of total PCBs) and PBDEs to a total of 4 congeners (to reach a total of 99% of the total measured content). Additionally, we administered this complex formulation by egg microinjection rather than bath exposure to assure consistent internal composition at the time of exposure to the chemical convulsant.

*p,p′*-DDE exposure of zebrafish embryos increases sensitivity of the fish to chemical convulsants. Our previous experiments indicated that this effect was apparent at bath exposure levels as low as 0.3 μM *p,p′-*DDE (Tiedeken and Ramsdell 2009). In the studies conducted here we used bath doses of 1.0 and 2.0 μM *p,p′*-DDE in the presence or absence of a contaminant formulation. The effects were determined in response to two chemical convulsants, PTZ and domoic acid. In addition to enhancing induced seizure behavior, *p,p′*-DDE also promotes a unique and readily observed head-shake behavior in response to the two chemical convulsants (Tiedeken and Ramsdell 2009). We observed no additional effects beyond those observed in *p,p′*-DDE–exposed embryos after coapplication of either the primary contaminant formulation or the complex contaminant formulation. This indicates that embryonic exposure to *p,p′*-DDE—and not exposure to co-occurring PCBs, β-HCH, PBDEs, and *trans*-nonachlor—is the primary contributor to greater sensitivity to domoic acid seizures at the completion of development.

### Relevancy of dosage of formulated mixture to fetal sea lion exposure levels

The embryonic exposures we used for the zebrafish experiments are based on a previously determined observable effect level of *p,p′-*DDE to enhance domoic acid–induced seizures, with the effective dose calibrated to wet-weight body concentration at the time of completed neurodevelopment. This dose corresponds to modeled levels of whole-body *p,p′*-DDE in full-term CSL fetuses based on 1991 data of the mothers consuming fish contaminated with 1,000 ng DDE/g wet weight (Connolly and Glaser 2002). When this dose is compared with actual wet-weight body concentrations of full-term CSLs, it corresponds to the highest levels of animals sampled in 2002, mid-range levels of animals sampled in 1996, and low exposure levels of animals sampled in 1972. The dosage of other organochlorines in our formulation is based on the ratio of individual congeners measured relative to mean *p,p′*-DDE values of 14 fetal sea lions. Hence, the total concentration of the complex contaminant mixture spans the range found in CINMS sea lions over a 30-year period and likely includes current concentrations found with the most susceptible population, that is, offspring of first-time pregnant animals, which are reported to have nearly a 10-fold higher concentration of DDTs and PCBs (Gilmartin et al. 1976).

### Interaction of PCBs with *p,p′*-DDE

PCBs are the most abundant co-contaminant with DDE in the sea lions of the CINMS. Originally identified to occur in both pregnant females and fetuses, the higher concentrations of both PCBs and DDTs found in females with aborted fetuses during a 1970 mortality event suggested the potential for an interactive effect (DeLong et al. 1973). An experimental study of this sea lion population 2 years later showed the same trend, with eight times higher DDT concentrations and four times higher PCB concentrations in those females with aborted fetuses than in females with normal term deliveries (Gilmartin et al. 1976). However, the identification of two pathogens, one of which was associated with reproductive failure in livestock, added a confounding factor that precluded implicating a role for PCBs or DDTs in the reproductive poisoning of these sea lions (Gilmartin et al. 1976).

PCBs have been associated with developmental complications in children and experimental animals. Substantial epidemiologic and experimental research in animals using PCBs has demonstrated the adverse effects of PCBs during development, with a primary effect of diminishing thyroid hormone levels (reviewed by Ulbrich and Stahlmann 2004). Epidemiologic studies have shown interaction between DDE and the four primarily occurring PCB congeners (118, 138, 153, and 180) to be correlated with measures of attention in early infancy (Sagiv et al. 2008). Developmental studies in rats comparing the commercial PCB mixture Arochlor 1254 with a formulation of PCBs, DDTs, and other persistent pesticides to match human blood composition lends insight to the interaction of these compounds (Bowers et al. 2004). Similarities between Arochlor 1254 and human blood formulation were best related to thyroid hormone–mediated actions, which are common for the PCB components of each mixture, whereas the presence of an organochloride/DDT component resulted in an overall increased toxicity and reproductive complications (Bowers et al. 2004). A differential action of DDT versus PCBs was noted in another study in which PCBs and HCH showed inverse correlation with thyroid hormone levels during pregnancy, whereas no correlation was found for DDT and thyroid hormone (Chevrier et al. 2008). Hence the different mode of action of PCBs may not affect seizure pathways modulated by developmental exposure to *p,p′*-DDE.

The lack of interaction we observed between PCBs and *p,p′*-DDE in increasing sensitivity to induced seizure behavior indicates that PCBs at the levels found in fetal sea lions do not contribute to this response. This response is similar to that found in a rodent study in which pregnant dams were given a PCB formulation to match human milk; the exposure did not alter *N*-methyl-d-aspartate receptors or long-term potentiation in the fetal hippocampus, but did reduce these end points in the occipital cortex (Altmann et al. 2001). The dominant PCB components of our complex contaminant formulation are similar to those in the human milk formulation. Accordingly, this PCB formulation lacks effect early in neurodevelopment (prior to synaptogenesis) on *N*-methyl-d-aspartate receptors, specifically their density, which mediate the excitotoxic effect of domoic acid in the region of the brain where domoic acid seizures originate.

### Interaction of PBDEs with *p,p′*-DDE

In contrast to PCBs, PBDEs levels have been increasing in wildlife and humans over the last two decades and, like PCBs, are found in high levels in the blubber of CSLs (Stapleton et al. 2006). Although PBDEs were not appreciably present at the time of the CSL mortality events of the 1970s, their presence over the last decade may play a role as other organochlorine contaminants are decreasing. Analysis of sea lion blubber from males stranded between 1993 and 2003 indicate that PBDEs probably reached their peak level (3,900 ng/g lipid) during this period. The levels of PBDE measured in fetal sea lions of the CINMS are 10 times lower (320 ng/g lipid) than those reported in male sea lions (Goldstein et al. 2009). The congener composition of both the male and fetal animals is very similar, with predominance of the penta-BDE congeners 47, 100, and 99. The 10-fold difference in concentrations between males and fetal sea lions is consistent with a similar magnitude of differences in PCBs and may be due to higher lifelong accumulation in males (Goldstein et al. 2009; Le Boeuf et al. 2002; Stapleton et al. 2006). PBDEs transfer with a maternal–fetal coefficient near 1, with levels found in human maternal and fetal blood averaging 33 ng/g lipid (Mazdai et al. 2003), about 10 times lower than those found in fetal sea lions.

PBDEs have neurotoxic effects comparable with those of PCBs in experimental animals, but supporting epidemiologic data are limited for the neurodevelopmental period (reviewed by Costa and Giordano 2007). PBDEs have shown adverse effects during neurodevelopment in mice, with a primary effect occurring during synaptogenesis, resulting in later-in-life changes in spontaneous behavior (hyperactivity) and impairments in learning and memory (Dencker and Eriksson 1998). These effects have been described for PBDE-99 and PBDE-47, two major components found in our formulation. PBDE-99 has been reported to have an additive effect with PCB-52, a fact not surprising given their similar effects on thyroid hormone levels and neurotoxicity (Dencker and Eriksson 1998). An absence of chemical convulsant–enhanced seizure behavior in our zebrafish study indicates that PBDEs at the concentrations reported in fetal sea lions, in combination with PCBs, demonstrate no interaction on *p,p′*-DDE–induced developmental neurotoxicity.

## Summary

We formulated a contaminant mixture of persistent pesticides, PCBs, and PBDEs to match the contaminant burden measured in fetal sea lions born prematurely during a recent domoic acid–associated mortality event. The addition of PCBs, HCH, chlordane, and PBDEs at concentrations that co-occur with DDT did not alter the effect of *p,p′-*DDE to enhance chemical-induced seizures in zebrafish. These results indicate that *p,p′-*DDE accounts for the major toxic activity of fetal sea lion contaminants that lead to greater sensitivity to domoic acid seizures.

## Figures and Tables

**Table 1:** Administration and composition of the different contaminant groups used in this study.

Treatment	Contaminants microinjected into embryo yolk (< 5 hpf)	Contaminants added to bath water of embryos (6–30 hpf)	Concentration of *p,p′-*DDE (other contaminants proportional)
Bath-water treatment			
Control	NA	0.1% DMSO vehicle	NA
1 μM *p,p′*-DDE	NA	*p,p′*-DDE	1 μM
Contaminant formulation	NA	*p,p′*-DDE, PCB-138, PCB-153, β-HCH, PBDE-47, *trans*-nonachlor	1 μM
2 μM *p,p′*-DDE	NA	*p,p′*-DDE	2 μM
2× Contaminant formulation	NA	*p,p′*-DDE, PCB-138, PCB-153, β-HCH, PBDE-47, *trans*-nonachlor	2 μM

Microinjection treatment			
Control	DMSO vehicle	0.1% DMSO vehicle	NA
1 μM *p,p′*-DDE	DMSO vehicle	*p,p′*-DDE	1 μM
Complex contaminant formulation with 1 μM *p,p′*-DDE	PCBs (153, 138, 118, 180, 101, 149, 99, 187, 110, 95, 105, 199, 52, 170, 87, 128, 151, 183, 49, 66), PBDEs (47, 100, 28, 99), β-HCH, *trans*-nonachlor	*p,p′*-DDE	1 μM
2 μM *p,p′*-DDE	DMSO vehicle	*p,p′*-DDE	2 μM
2× Complex contaminant formulation with 2 μM *p,p′*-DDE	PCBs (153, 138, 118, 180, 101, 149, 99, 187, 110, 95, 105, 199, 52, 170, 87, 128, 151, 183, 49, 66), PBDEs (47, 100, 28, 99), β-HCH, *trans*-nonachlor	*p,p′*-DDE	2 μM

NA, not applicable.

**Table 2:** Ratios of contaminant levels reported in fetal CSLs and corresponding formulations used for bath exposure of zebrafish embryos.

Contaminant group in sea lion pups	Average total (ng/g ww)^a^	Dominant congeners	Congener (% of total)^a^	Calculated congener concentration (ng/g ww)	Ratio of congener to *p,p′*-DDE	Contaminant formulation (μM)	2× Contaminant formulation (μM)
∑DDTs	1,700	*p,p′*-DDE	99	1683.000	1.000	1.000	2.000
∑PCBs	360	PCB-153	27	97.200	0.058	0.058	0.116
∑PCBs	360	PCB-138	19	68.400	0.041	0.041	0.081
∑HCHs	22	β-HCH	100	22.000	0.013	0.013	0.026
∑PBDEs	140	PBDE-47	83	116.200	0.069	0.069	0.138
∑Chlordanes	63	*trans*-Nonachlor	80	50.400	0.030	0.030	0.060

Abbreviations: ∑, sum of compounds, ww, wet weight.

a Values interpreted from Goldstein et al. (2009).

**Table 3:** Ratios of contaminant congener composition in fetal sea lions and corresponding formulation of mixture used for microinjection of zebrafish embryos.

Contaminant groups in sea lion pups	Average total (ng/g ww)^a^	Dominant congeners	Congener (% of total)^a^	Calculated congener concentration (ng/g ww)	Ratio of congener to *p,p′*-DDE	Nanograms per egg (*p,p′*-DDE = 7.49 ng)^b^	Complex contaminant formulation (ng/nL DMSO)	2× Complex contaminant formulation (ng/nL DMSO)
∑ DDTs	1,700	*p,p′* DDE	99	1,683	1	7.499	Bath exposure	Bath exposure
∑ HCHs	63	β-HCH	100	22.00	0.01307	0.098	0.098	0.196
∑ Chlordanes	22	*trans*-Nonachlor	80	50.40	0.02995	0.225	0.225	0.449
∑ PCBs	360							
		PCB-153	21.0	75.60	0.04492	0.337	0.337	0.674
		PCB-138	15.0	54.00	0.03209	0.241	0.241	0.481
		PCB-118	7.5	27.00	0.01604	0.120	0.120	0.241
		PCB-180	6.5	23.40	0.01390	0.104	0.104	0.209
		PCB-101	6.0	21.60	0.01283	0.096	0.096	0.192
		PCB-149	5.5	19.80	0.01176	0.088	0.088	0.176
		PCB-99	4.8	17.28	0.01027	0.077	0.077	0.154
		PCB-187	4.0	14.40	0.00856	0.064	0.064	0.128
		PCB-110	3.5	12.60	0.00749	0.056	0.056	0.112
		PCB-95	3.0	10.80	0.00642	0.048	0.048	0.096
		PCB-105	2.2	7.92	0.00471	0.035	0.035	0.071
		PCB-199	2.2	7.92	0.00471	0.035	0.035	0.071
		PCB-52	2.0	7.20	0.00428	0.032	0.032	0.064
		PCB-170	2.0	7.20	0.00428	0.032	0.032	0.064
		PCB-87	1.9	6.84	0.00406	0.030	0.030	0.061
		PCB-128	1.8	6.48	0.00385	0.029	0.029	0.058
		PCB-151	1.6	5.76	0.00342	0.026	0.026	0.051
		PCB-183	1.5	5.40	0.00321	0.024	0.024	0.048
		PCB-49	1.4	5.04	0.00299	0.022	0.022	0.045
		PCB-66	1.4	5.04	0.00299	0.022	0.022	0.045
∑ PBDEs	140							
		PBDE-47	70	98.00	0.05823	0.437	0.437	0.873
		PBDE-100	20	28.00	0.01664	0.125	0.125	0.250
		PBDE-99	8	11.20	0.00665	0.050	0.050	0.100
		PBDE-28	2	2.80	0.00166	0.012	0.012	0.025

Abbreviations: ∑, sum of compounds; ww, wet weight.

a Values interpreted from Goldstein et al. (2009).

b Value measured from Tiedeken and Ramsdell (2009).

**Table 4:** Effect of exposure to 0.36 mM domoic acid on behavior of 7 dpf zebrafish larvae preexposed as embryos to *p*,*p′*-DDE alone or in a contaminant formulation.

	Class 1		Class 2		Class 3	
Treatment	Individuals responding (%)	No. of responses	Individuals responding (%)	No. of responses	Individuals responding (%)	No. of responses
Control/vehicle	88	79	63	26	75	28
1 μM *p,p′-*DDE	100	87	90	51	70	46
Contaminant formulation	100	82	78	59	78	20
2 μM *p,p′-*DDE	100	95	100	99	80	58
2× Contaminant formulation	100	57	100	93	100	67

Responses are grouped by classes of perceived increased severity (Class 1, 2, and 3). The time progression for the data is available in Supplemental Material, Table 1 (doi:10.1289/ehp.0901301).

**Table 5:** Effect of exposure to 0.36 mM domoic acid on behavior of 7 dpf zebrafish larvae preexposed as embryos to *p*,*p′*-DDE alone or egg microinjection of complex contaminants.

	Class 1		Class 2		Class 3	
Treatment	Individuals responding (%)	No. of responses	Individuals responding (%)	No. of responses	Individuals responding (%)	No. of responses
Control/vehicle	83	22	75	22	60	11
1 μM *p,p′-*DDE	100	64	100	91	70	34
Complex formulation + 1 μM *p,p′*-DDE	91	64	100	90	64	58
2 μM *p,p′-*DDE	91	68	100	160	91	87
2× Complex formulation + 2 μM *p,p′*-DDE	89	60	100	142	100	72

Responses are grouped by classes of perceived increased severity (Class 1, 2, and 3). The time progression for the data is available in Supplemental Material, Table 2 (doi:10.1289/ehp.0901301).
